# Orthogeriatric co-management: differences in outcome between major and minor fractures

**DOI:** 10.1007/s00068-022-01974-3

**Published:** 2022-04-28

**Authors:** Andreas Wiedl, Stefan Förch, Annabel Fenwick, Leonard Lisitano, Timon Röttinger, Thilo Nachbaur, Alexander Otto, Edgar Mayr

**Affiliations:** Abteilung für Unfallchirurgie, Orthopädie, Plastische und Handchirurgie, Universitätsklinikum Augsburg, Stenglinstraße 2, 86156 Augsburg, Germany

**Keywords:** Orthogeriatric co-management, Fragility fractures, Mobility, Barthel index, Mortality, Place of residence

## Abstract

**Purpose:**

Literature shows that orthogeriatric co-management improves the outcomes of patients with hip fractures. Corresponding research with more diverse fragility fracture groups is lacking.

Therefore, an examination was performed prospectively as a 2 year-follow-up on an orthogeriatric co-managed ward, comparing relevant outcome parameters for major and minor fragility fractures.

**Methods:**

All patients treated on an orthogeriatric co-managed ward from February 2014 to January 2015 were included and their injuries, orthogeriatric parameters such as the Barthel Index (BI), Parker Mobility Score (PMS) and place of residence (POR). Patients were separated into two groups of either immobilizing major (MaF) or non-immobilizing minor (MiF) fractures. 2 years later, a follow-up was conducted via telephone calls and questionnaires mailed to patients and/or their relatives.

**Results:**

740 (574 major vs. 166 minor injuries) patients were initially assessed, with a follow-up rate of 78.9%. The in-house, 1-year, and 2-year-mortality rates were 2.7, 27.4, and 39.2%, respectively. Mortality was significantly higher for MaF in the short term, but not after 2 years. On average, during the observation period, patients regained their BI by 36.7 points (95% CI: 33.80–39.63) and PMS was reduced by 1.4 points (95% CI: 1.16–1.68). No significant differences were found in the readmission rate, change in BI, PMS or POR between the MaF and MiF groups.

**Conclusion:**

The relevance of orthogeriatric treatment to improving functional and socioeconomic outcomes was confirmed. The similarity of the results from both fracture groups emphasizes the need for a multidisciplinary approach also for minor fractures.

**Supplementary Information:**

The online version contains supplementary material available at 10.1007/s00068-022-01974-3.

## Introduction

On average, 2.7 million fragility fractures occur annually in Europe alone, while increasing numbers of these fractures increase the burden on health care systems and health care providers [1]. The orthogeriatric patient requires a more complex treatment approach than a younger patient in the trauma unit [2]. Multimorbidities, frailty, cognitive impairment, immobility, reduced capacity for self-care, and organizational issues (e.g., a change of accommodation) are some of the main issues driving these patients’ complex needs [2]. To address these and provide complementary treatment for orthogeriatric patients, orthogeriatric co-management was established [2–5]. It involves a multi-professional team of geriatric and orthopedic specialists, physio- and ergotherapists, and social helpers that build the foundation of a multidimensional approach [3]. Several studies have already shown that orthogeriatric co-management improves patients’ mortality and recovery of function as well as socioeconomic aspects such as place of residence, the need for care, and retention of accommodation [6–10]. These outcome parameters have consequently been evaluated for hip fracture patients as hip fractures are the most common fragility fractures associated with age. Nevertheless, in a more holistic view of today’s orthogeriatric treatment, a multitude of injuries, including forearm, humeral, vertebral, rib, and pelvic fractures are of concern. Although some investigations examine these other fragility fractures of the elderly [11, 12], the literature is still lacking an adequate overview of co-management’s impact on them and a comparison of major and minor fractures according to their immobilizing or non-immobilizing character, respectively.

In this investigation, a 2 year follow-up was performed, focusing on orthogeriatric patients who received co-management. The causes of admission were stratified into two groups, major and minor injuries. Fracture-independent and -dependent functions, socioeconomic outcomes, and survival were evaluated. Our objective was to examine, on one hand, the importance of orthogeriatric co-management for the mentioned outcome parameters and, on the other hand, whether outcomes in this context differ between major and minor fractures.

## Methods

An entire cohort of patients treated on a co-managed orthogeriatric ward during 1 year was assessed, from February 2014 to January 2015. The institutional review board in charge approved of this study (7/11192) and the patients and/or their legal guardians gave their informed consent. The patients were treated for different fragility fractures. Those admitted for non-fracture-associated causes were excluded. An in-house score was used for screening admission adequacy to the orthogeriatric ward: one point each for age greater than 75 years, auditory impairment, visual impairment, polypharmacy (≥ 5 medications), dementia, frequent falling, and sarcopenia. Two points were given for impaired mobility or the need for aids like crutches or wheeled walkers. A total of four or more points qualified patients for admission. The exclusion criteria were immobility and severe dementia (determined by Mini-Mental State Exam scores of < 10 points and individual assessment).

Each patient’s injury and therapy were recorded. Several assessments and socioeconomic parameters were included.

Included data:

- Barthel Index [13] (BI) on admission, discharge, and in the follow-up.
  - BI evaluates the patients’ capacity for self-care in eating, sitting up and moving to another seat, washing, using the toilet, showering, mobility, climbing stairs, dressing and undressing, and urinary and fecal incontinence.
  - A maximum of 100 points is possible, describing a patient who is completely capable in every category. 0 points indicate complete dependence on assistance.
- Parker Mobility Score [14] (PMS) before admission and in the follow-up.
  - PMS scores range from 0 (no mobility) to 9 (highest mobility) in the three modalities of walking inside the home, outside the home, and shopping, respectively,
    - 3 points are allotted for unrestricted walking.
    - 2 for walking with aids.
    - 1 for walking with the help of another person.
    - 0 for being unable to walk at all.
- Readmission rate (general and due to falls) until the follow-up.
- Care level (CL) before admission and in the follow-up.
  - Care level at assessment time was defined by the German Code of Social Law XI § 15:
    - No care level: the patient is totally capable of self-care or needs minimal help.
    - Care level 1: the patient needs > 90 min of support per day.
    - Care level 2: the patient needs > 180 min of support per day.
    - Care level 3: the patient needs > 300 min of support per day.
- Place of residence (POR) before admission and in the follow-up.
  - We differentiated among patients living.
    - At home.
    - In sheltered housing.
    - In nursing homes.
- Personal assistance needed by home-dwelling patients before admission and in the follow-up.
- Personal (patient’s and/or relative’s) evaluation of the situation at follow-up.
- In-house, 1- and 2-year-mortality.
- Charlson Comorbidity Index (CCI) for each group, subdivided into [15]
  - CCI < 4 points.
  - CCI ≥ 4 points.

### Therapy

Every patient received physiotherapy twice, ergotherapy once a day, and orthogeriatric co-management by both an orthopedic and geriatric specialist. Daily team briefings among doctors, physio- and ergotherapists, social service workers, and neuropsychologists were performed to discuss patients and their perspectives. “Complex early geriatric rehabilitation” (CEGH) required an inpatient stay of at least 16 days. Not every patient was included in CEGH; nevertheless, every patient benefitted from the same therapeutic modalities. The only difference in CEGH was a longer inpatient stay.

Primary hip fractures were treated surgically with either intramedullary nail osteosynthesis (AO31A1-3) or primary hip hemi- or total arthroplasty (AO31B1-3).

Thoracolumbar osteoporotic vertebral fractures were treated using a decisional algorithm considering the fracture morphology, pain, mobility, further vertebral collapse, and bone density, resulting in a ratio of 56% conservative to 44% operative treatment.

Pelvic ring fractures underwent conservative treatment, as all in the cohort were estimated stable at assessment time. Acetabular fractures were addressed by either plating or endoprosthesis.

Humeral fractures were treated osteosynthetically with intramedullary nails or plates and forearm fractures with volar plating. Of these patients, 92% of those with humeral fractures and 75% of those with forearm fractures were treated surgically and the remaining cases were treated conservatively.

Rib fractures were treated conservatively with breathing exercises and analgesia.

Cervical spine fractures were evaluated for stability and treated surgically through anterior and/or dorsal fixation if they were determined to be unstable. Thus, 65% of cervical spine injuries were treated surgically.

### Injury stratification

To facilitate comparison, we divided the patients into two groups of major, mostly immobilizing injuries and minor, mostly non-immobilizing injuries.

**Fig. 1:**
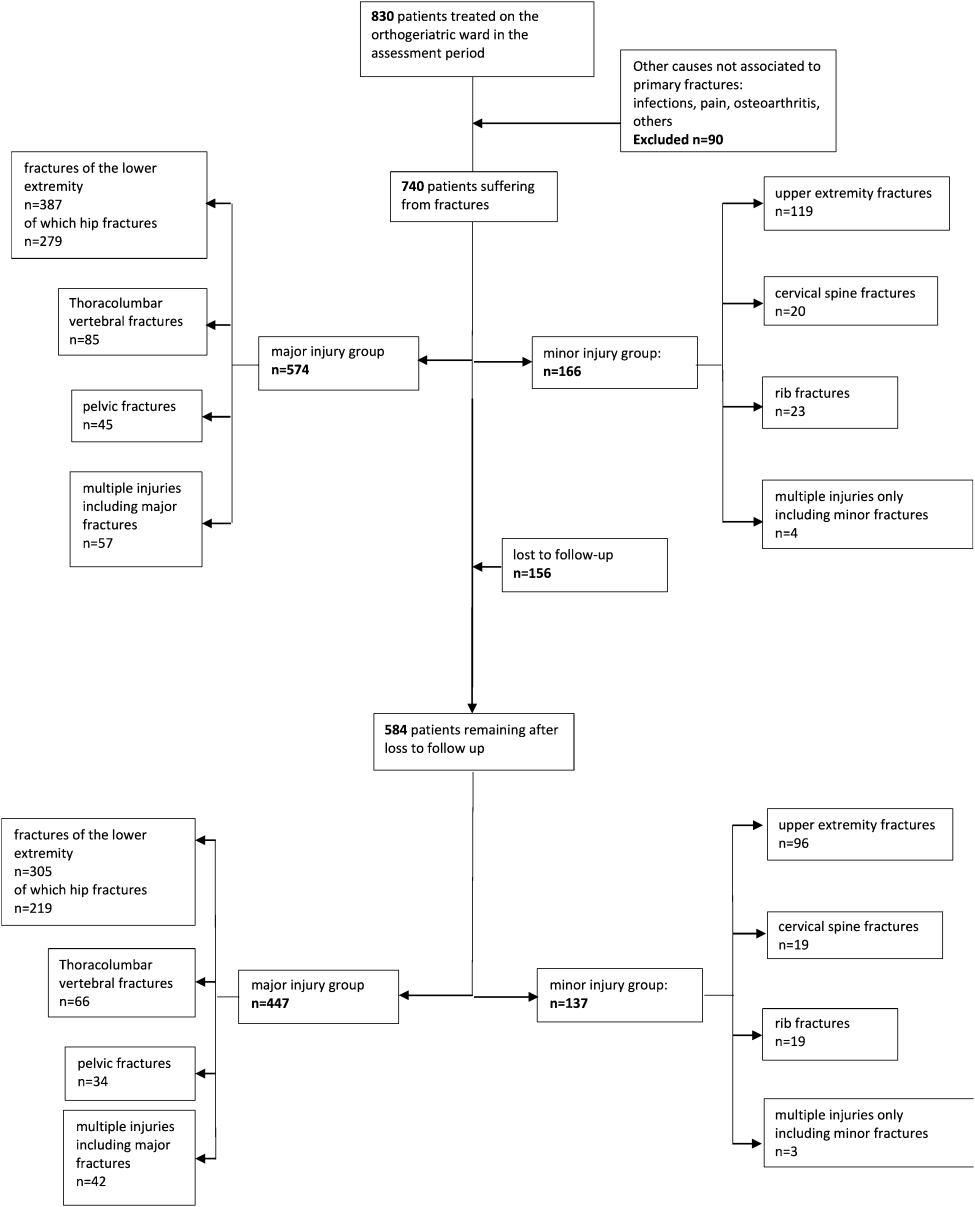
Patient numbers initially and at follow-up for each fracture group

Figure 1 shows the initial and adjusted (for loss to follow-up) distribution and case numbers for each injury and cause of admission. Injuries to the lower extremities (including hip fractures), thoracolumbar spine, and pelvis were considered major, while those to the upper extremities, cervical spine, and ribs were labeled minor fractures. Infections or other causes of admission (such as osteoarthritis or pain) were excluded from our assessment.

### Follow-up

Patients and/or their relatives were contacted approximately 2 years after discharge with mailed questionnaires to obtain information about their actual BI, PMS, readmission rate, care level, POR, need for assistance, subjective impression of the situation, and whether the patient had passed away. In cases of no response, in an attempt for a telephone interview, a maximum of five telephone calls were made.

### Statistical analysis

The statistical work was performed with SPSS Version 1.0.0.1461 (IBM Corp., Armonk, New York, USA). Normal distribution was evaluated with Kolmogorov–Smirnov tests. The analyses revealed no normal distribution for any parameter. Therefore, Mann–Whitney *U*, Kruskal–Wallis, and Wilcoxon tests were performed for non-paired and paired samples. Linear regression was used to screen for confounding effects concerning LOS and CEGH.

Categorical variables such as POR, care level, and mortality were evaluated for differences with chi-squared tests. To determine the hazard ratio (HR), we employed the Cox regression model for proportional hazards. A *p*-value of 0.05 was considered significant. Pairwise deletion was used to handle any missing data.

We indicated total counts for respective case numbers, fractions in percentages, and continuous variables with means and standard deviations. For variable-wise respective differences from admission to follow-up, we stated means and 95% confidence intervals.

### Potential biases

A channeling and treatment bias can be presumed considering the admission criteria to the orthogeriatric ward. Nevertheless, the admission score was intended to select a more homogenous patient cohort to reduce confounding effects and allow the comparison of major and minor fractures. The loss to follow-up was minimized by implementing telephone contact interviews to conduct our questionnaire.

## Results

A total of 740 patients suffering from major or minor fractures, as shown in Fig. 1, were assessed during the in-hospital period.

Of these, 584 patients (78.9%) were included in the follow-up and the mean follow-up time was 617.7 ± 187.16 days. Although major fractures exhibited a subtly higher loss to follow-up than minor fractures did, the difference was insignificant (*p* = 0.380) (Tables 3, 4). 229 patients were deceased after 2 years, leaving 355 cases for the evaluation of further parameters. Due to isolated deficits in the documentation of several assessment items, further assessment rate losses occurred, as stated in the supplemental material for each variable.

In this study, 574 major and 166 minor injuries were assessed. The patients’ mean age was 84.5 ± 6.59 years. It did not differ much nor significantly between the major (84.5 ± 6.67) and minor (84.6 ± 6.33) groups (*p* = 0.950). There was no significant difference in the gender distribution between the groups; 562 women and 178 men were treated in total (*p* = 0.918). The CCI distribution showed a significantly higher proportion of patients with a CCI > 4 in the major fracture group (Table 1) (*p* < 0.001). The mean length of stay (LOS) overall was 15.8 ± 7.78 days. The LOS was significantly lower for those with minor fractures, at a mean duration of 13.3 ± 7.99 days, than for those with major fractures, at a mean duration of 16.6 ± 7.56 days (*p* < 0.001). Significantly more patients in the major group received CEGH (51.0%) than in the minor group (36.2%) (*p* = 0.002). Nevertheless, after linear regression, we still observed a significant prolonged stay of 1.8 ± 0.64 days after major fractures.

**Table 1:** Characteristics and distribution of all patients and major and minor fracture group Total numbers, means standard ± deviation and percentages

	All patients	Major fractures	Minor fractures	*p* values for significant differences in between major- and minor group
N initial	740	574	166	
N at follow-up	584	447	137	
Age (years)	84.5 ±6.59	84.5 ±6.67	84.6 ±6.33	0.950
Gender distribution female: male (percentage)	75.9%: 24.1%	75.8%: 24.2%	76.5%: 23.5%	0.918
CCI				
<4	61.1%	57.6%	73.5%	<0.001
≥4	38.9%	42.4%	26.5%	
BI on admission	25.2 ± 16.51	23.4 ± 15.34	31.5 ± 18.74	<0.001
BI on discharge	42.5 ± 19.96	41.4 ± 19.67	46.7 ± 20.50	0.008
BI in follow-up group	64.8 ± 30.65	62.4 ±31.21	71.9 ± 27.98	0.013
PMS before admission	5.2 ± 2.61	5.1 ± 2.57	5.4 ± 2.72	0.371
PMS in follow-up group	4.5 ± 2.62	4.3 ± 2.56	5.0 ± 2.76	0.051
LOS (days)	15.8 ± 7.78	16.6 ± 7.56	13.3 ± 7.99	<0.001
Admission from (ratios in percentage)				
Home dwellers	75.5%	74.2%	80.1%	0.444
Sheltered housing	5.0%	5.0%	5.0%	
Nursing home	19.5%	20.8%	14.9%	
POR at follow-up (ratios in percentage)				
Home	67.1%	63.7%	76.9%	
Sheltered housing	6.2%	6.1%	6.6%	
Nursing home	26.6%	30.6%	16.5%	
Care level before admission (ratios in percentage)				
None	59.8%	57.3%	67.1%	0.012
1	27.0%	27.3%	26.1%	
2	11.7%	13.4%	6.8%	
3	1.5%	2.0%	0%	
Care level at Follow-up(ratios in percentage)				
None	35.5%	32.4%	44.3%	
1	35.5%	35.2%	36.4%	
2	23.1%	24.9%	18.2%	
3	5.9%	7.5%	1.1%	

### Daily living activities and mobility

**Fig. 2:**
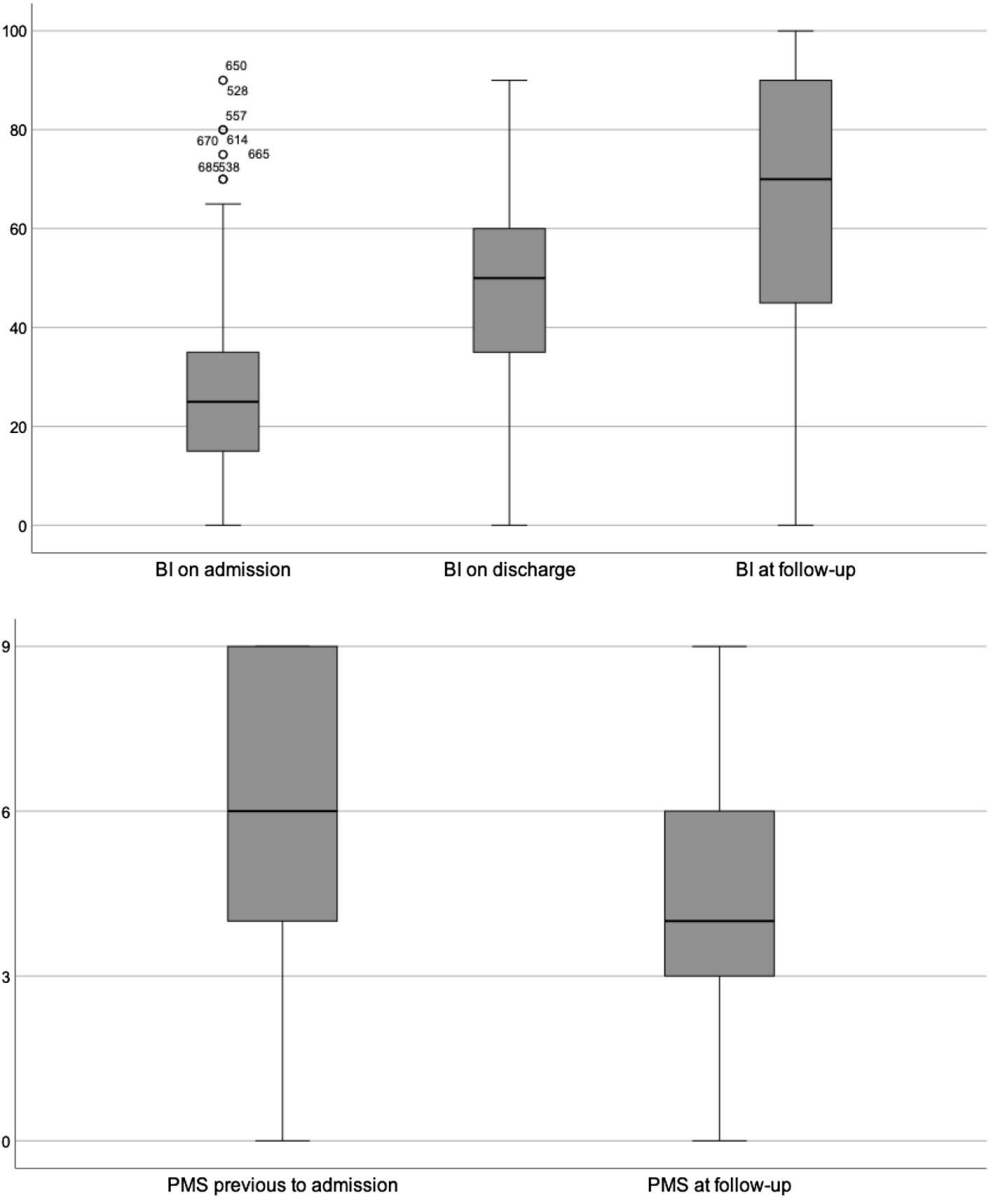
The course of general Barthel Index and Parker Mobility Scores

**Fig. 3:**
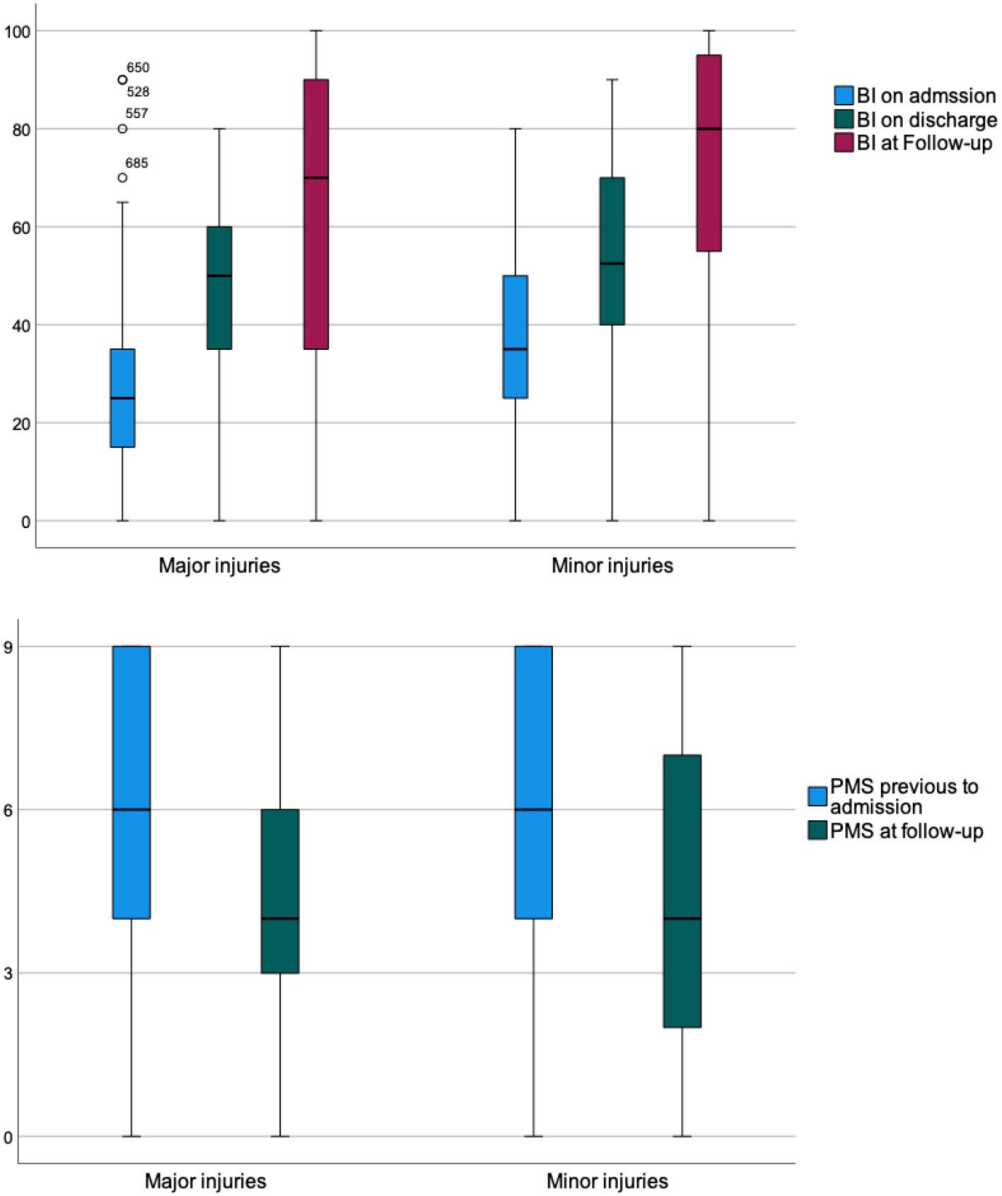
The course of group-specific Barthel Index and Parker Mobility Scores

Mean BI on admission was 25.2 ± 16.51 for all patients; the values for patients in the minor group (31.5 ± 18.74) were significantly higher than those for patients in the major group (23.4 ± 15.34) (*p* < 0.001) (Table 1). The BI increased significantly in all groups from admission to discharge and from discharge to follow-up, with a total recovery of 36.7 (33.80–39.63) (Tables 2, 3, 4, Figs. 2, 3). The BI on discharge was 42.5 ± 19.96 overall, 41.4 ± 19.67 for major fractures, and 46.7 ± 20.50 for minor fractures (*p* = 0.008). At follow-up, a mean overall BI of 64.8 ± 30.65 was observed, again with significant differences between the major (62.4 ± 31.21) and minor (71.9 ± 27.98) fracture groups (*p* = 0.013). Nevertheless, for both groups, the overall BI recovery from admission to follow-up was not significantly different (major fractures: 36.9 [33.44–40.32]; minor fractures: 36.2 [30.69–41.78]) (Tables 2, 3, 4, Figs.2, 3) (*p* = 0.811).

**Table 2:** All patients‘ outcome

	Means (95% CI)	Wilcoxon-test 2-sided *p*-value
Change of BI on admission to BI on discharge	18.1 (16.77–19.39)	<0.001
Change of BI in follow-up to BI on discharge	17.7 (14.85–20.52)	<0.001
Change of BI in follow-up to BI on admission	36.7 (33.80–39.63)	<0.001
Change of PMS in follow-up to PMS before trauma	− 1.4 (− 1.68 to − 1.16)	<0.001
	Percentages (n)	
Follow-up rate	78.9% (584)	
Higher care level	36.7% (125/341)	
Different POR		
In general	16.2% (56/345)	
For previous home dwellers	1.1% (50/277)	
Need for more assistance at home	50.2% (101/201)	
Subjective impression of situation		
Much worse	3.4% (11)	
Worse	33.6% (108)	
Same	25.5% (82)	
Better	32.1% (103)	
Much better	5.3% (17)	
Rehospitalization		
Due to fracture or falls	12.4% (40/323)	
In-House-mortality	2.7% (20/740)	
1 year-mortality ratio	27.4% (160/584)	
2 year-mortality	39.2% (229/584)

**Table 3:** Outcome after major injuries

	Means (95% CI)	Wilcoxon-test 2-sided *p*-value
Change of BI on admission to BI on discharge	18.8 (17.32–20.28)	<0.001
Change of BI in follow-up to BI on discharge	16.3 (12.96–19.54)	<0.001
Change of BI in follow-up to BI on admission	36.9 (33.44–40.32)	<0.001
Change of PMS in follow-up to PMS before trauma	− 1.5 (− 1.80 to − 1.20)	<0.001
	Percentages (n)	
Follow-up rate	77.9% (447)	
Higher care level	39.5% (100/253)	
Different POR		
In general	17.1% (44/257)	
For previous home dwellers	19.5% (39/200)	
Need for more assistance at home	53.5% (76/142)	
Subjective impression of situation		
Much worse	4.2% (10)	
Worse	36.0% (85)	
Same	21.6% (51)	
Better	33.5% (79)	
Much better	4.7% (11)	
Rehospitalization		
In general	46.3% (111/240)	
Due to fracture or falls	12.5% (30/240)	
In-house-mortality	3.5% (20/574)	
1 year mortality	29.8% (133/447)	
2 year mortality	40.9% (183/447)

**Table 4:** Outcome after minor injuries

	Means (95% CI)	Wilcoxon-test 2-sided *p*-value
Change of BI on admission to BI on discharge	15.4 (12.61–18.26)	<0.001
Change of BI in follow-up to BI on discharge	22.1 (16.48–27.68)	<0.001
Change of BI in follow-up to BI on admission	36.2 (30.69–41.78)	<0.001
Change of PMS in follow-up to PMS before trauma	− 1.2 (− 1.68 to − 0.70)	<0.001
	Percentages (n)	
Follow-up rate	82.5% (137)	
Higher care level	28.4% (25/88)	
Different POR		
In general	13.6% (12/88)	
For previous home dwellers	14.3% (11/77)	
Need for more assistance at home	42.4% (25/59)	
Subjective impression of situation		
Much worse	1.2% (1)	
Worse	27.1% (23)	
Same	36.5% (31)	
Better	28.2% (24)	
Much better	7.1% (6)	
Rehospitalization		
In general	39.8% (33/83)	
Due to fracture or falls	12.0% (10/83)	
In-house-mortality	0% (0/166)	
1 year mortality	19.7% (27/137)	
2 year mortality	33.6% (46/137)

The PMS overall at admission was 5.2 ± 2.61. It was comparable between the two fracture groups (5.1 ± 2.57 for major and 5.4 ± 2.72 for minor fractures; *p* = 0.371). A significant mean decline of PMS was observed in all groups at follow-up (Tables 2, 3, 4, Figs.2, 3). The decline in each fracture group was not significantly different (major fractures: − 1.5 [− 1.80 to − 1.20]; minor fractures: − 1.2 [− 1.68 to − 0.70]) (*p* = 0.230). For all patients at follow-up, we calculated a PMS of 4.5 ± 2.62, or 4.3 ± 2.56 for those in the major group and 5.0 ± 2.76 for those in the minor group (*p* = 0.051).

### Rehospitalization

A total of 144 patients (44.6%) had to attend a hospital at least once between discharge and the follow-up assessment. Of these, 40 (12.3%) were readmitted due to falls or fractures.

No significant differences were found between the major and minor group concerning rehospitalization. This was true for general reasons for readmission (major: 46.3% vs. minor: 39.8%, *p* = 0.370) and for fracture- or fall-associated reasons (major: 12.5% vs. minor 12.0%, *p* = 0.914).

### Care level and place of residence (POR)

**Fig. 4:**
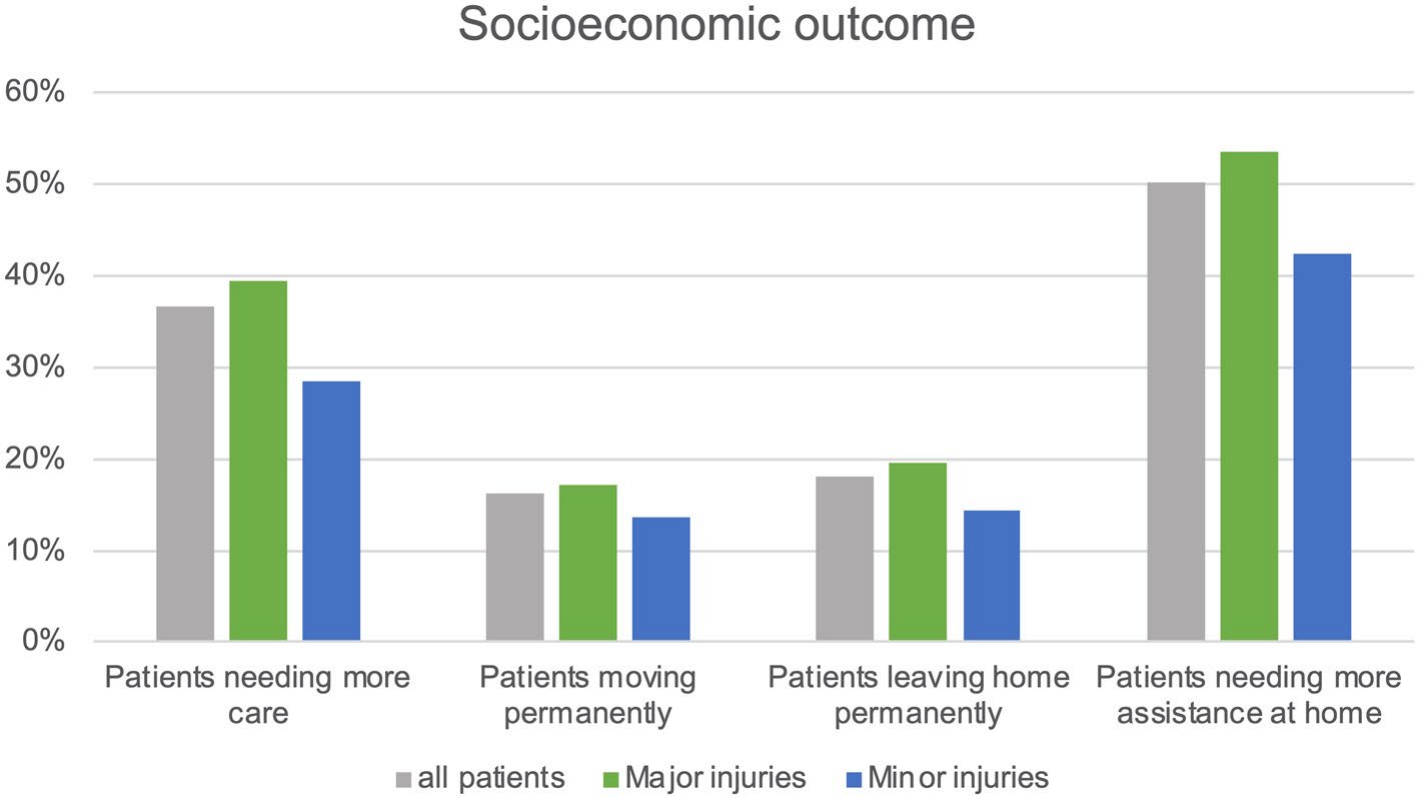
Overview of socioeconomic outcomes: patients needing more care, moving permanently, leaving home permanently, or needing more assistance at home

Significant numbers of patients in each group had been graded at higher care levels by the follow-up (*p* < 0.001) (Table 1). In general, 36.7% were reclassified at higher care levels. Significantly more patients suffering from major fractures (39.5%) experienced upgraded care levels compared to those suffering from minor fractures (28.4%) (*p* = 0.012) (Fig. 4).

In total, 75.5% of patients had lived at home before admission, while 5.0% lived in sheltered housing and 19.5% lived in nursing homes. A significant number of patients lived in different accommodations at follow-up (*p* = 0.014). Overall, 16.2% of all patients had to move permanently after discharge and 18.1% of previously home-dwelling patients had to leave their homes.

In the major fracture group, there was also a significant change in POR between admission and follow-up (*p* = 0.008). Overall, 17.1% of patients had to move and 19.5% that had lived at home had to leave their homes permanently.

The minor fracture group experienced fewer changes in POR. Only slightly more patients were living in nursing homes at follow-up (16.5%) than on admission (14.9%) and these changes were insignificant (*p* = 0.801). In total, 13.6% of patients suffering from minor fractures had a different POR at follow-up and 14.3% of home dwellers had to leave their homes permanently.

Among all patients still able to live at home, 50.2% needed more assistance in general; 53.5% of those in the major and 42.4% of those in the minor group.

In a pairwise comparison of the groups, we could detect neither significant differences in the total change of patients’ accommodations (*p* = 0.444), nor the moving frequency of previously home-dwelling patients (*p* = 0.312), nor the need for more assistance at home (*p* = 0.150).

**Fig. 5:**
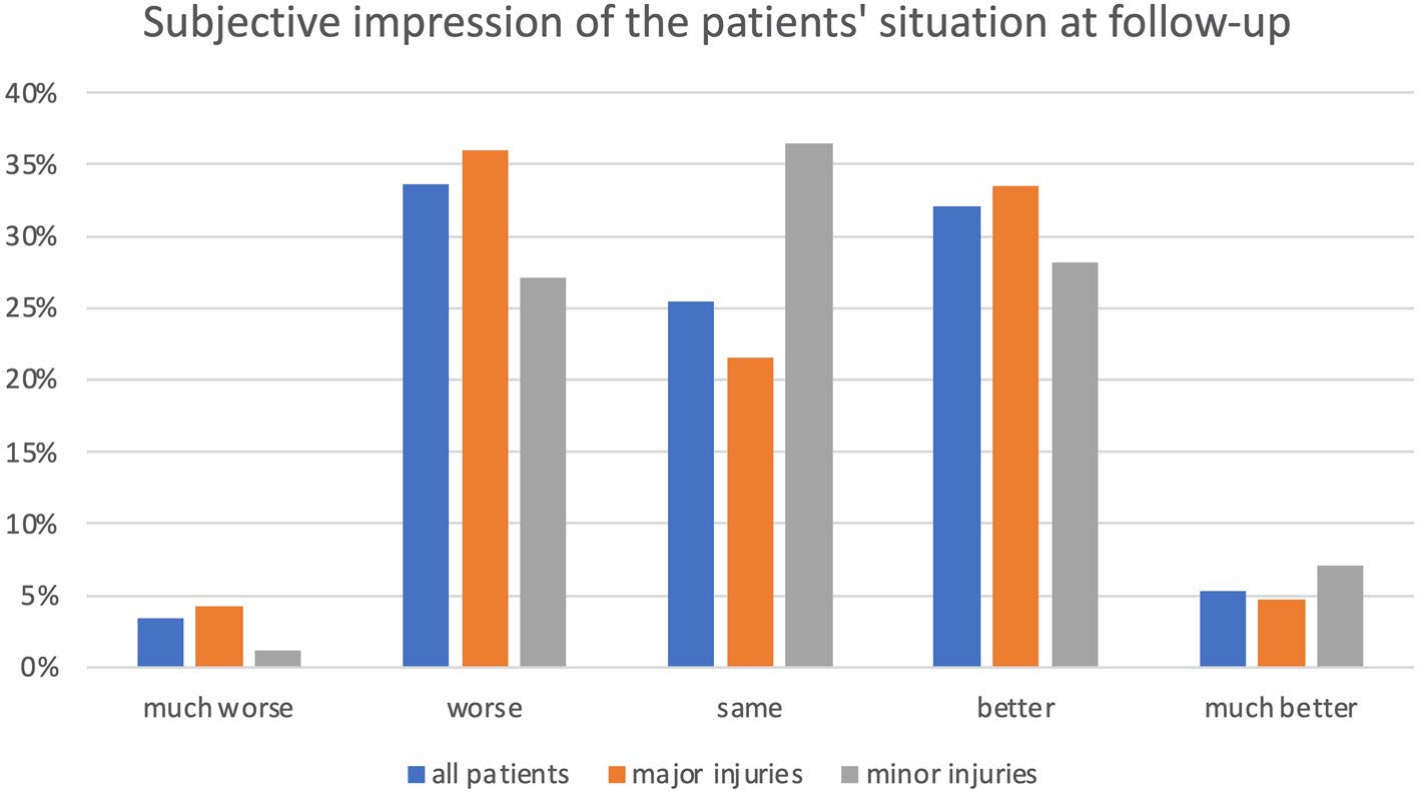
Patients’ and relatives’ subjective evaluation at follow-up

Concerning patients’ or relatives’ subjective evaluation of the situation at follow-up, 25.5% reported no changes, 32.1% reported a “better” impression, and 33.6% reported a “worse” impression. “Much better” or “much worse” statements were only reported in 5.3 and 3.4% of cases, respectively. Significantly more patients in the minor group (71.8%) reported at least a “same” or “better” statement at follow-up than those in the major group (59.8%) (*p* = 0.0437) (Tables 2, 3, 4) (Fig. 5).

### Mortality

**Fig. 6:**
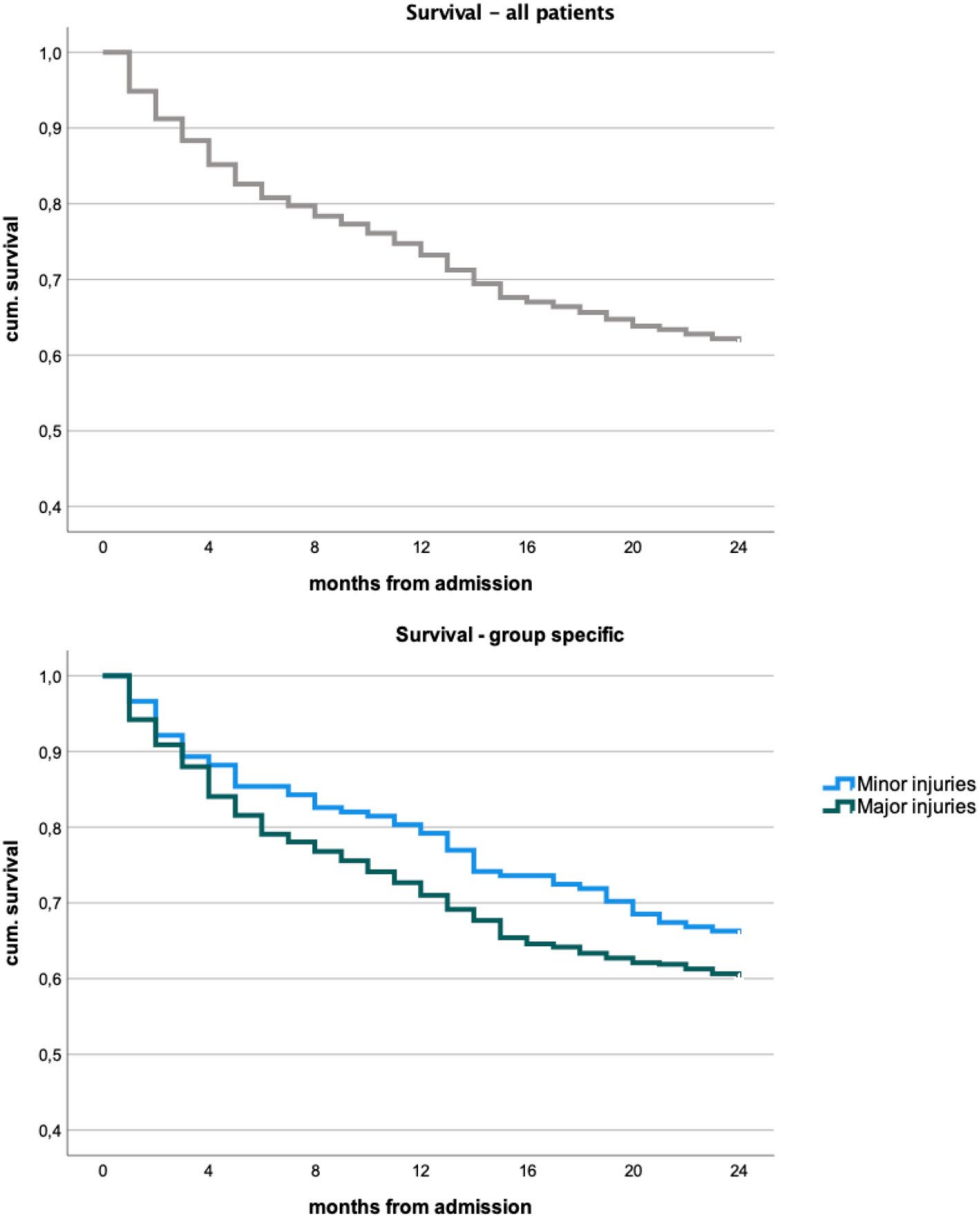
General and group-specific survival curves

A total of 20 patients were deceased during the in-hospital stay, all of whom had suffered from major fractures, resulting in an in-house mortality rate of 2.7%. After adjusting for loss to follow-up, a total 1- and 2-year-mortality rate of 27.4% (*n* = 160) and 39.2% (*n* = 229) was observed (Table. 2, Fig. 6). Patients in the major group had a distinctly but not significantly higher risk of death during the observation period with a hazard ratio of 1.3 (95% CI: 0.95–1.81) (*p* = 0.097). While in-house and 1-year-mortality were significantly higher in the major group (*p* = 0.011 and 0.022, respectively), 2-year-mortality showed no significant difference (*p* = 0.134) (Tables 3, 4, Fig. 6).

## Discussion

Overall, we found a significant recovery of the activities of daily living but a slight, significant decline in mobility. Also, approximately one-third of all patients were classified as being in higher need of care and approximately one-fifth of the home dwellers had to leave their homes.

By comparing major and minor injuries, no significant differences were found between groups concerning changes in function, accommodation, domestic help, and 2-year-mortality. As the only remarkable differences, LOS and the need for care at follow-up were significantly higher and patients’ and relatives’ subjective satisfaction were significantly lower after major fractures. Although in-house and 1-year-mortality was significantly higher for major fractures, this difference vanished after 2 years. Notably, significantly more patients in the major group had higher morbidity scores than those in the minor group, which suggests even more comparable outcomes, all else being equal.

Mobility and the activities of daily living are two of the major outcome parameters worth evaluating in geriatric patients who have experienced trauma [16].

The regain and preservation of function are major aspects of orthogeriatric treatment. Stenvall et al. and Prestmo et al. have shown superior development of functionality and mobility after multidisciplinary treatment compared with conventional treatment in randomized, controlled studies [17, 18]. In our study, the increased BI score, as a marker of functional regain, was remarkable, although we must respect that the initial baseline was assessed directly after admission and the fracture event.

The amelioration of BI from discharge to follow-up was comparable to the initial change from admission to discharge. This supports the profound importance of acute rehabilitation during the in-hospital course, as well as the necessity of further rehabilitation after discharge. In the context of orthogeriatric treatment, Neuerburg et al. also described significant improvements in the activities of daily living for hip-fracture patients after 1 year [9]. Gosch et al. describe significant increases in BI for their long-term care residents independent of fracture from day 5 to a 3 month follow-up [7]. Another study supports these findings, reporting an average increase of BI from discharge to 1 year follow-up of 23.42 points (hip fracture patients that underwent inpatient rehabilitation after acute treatment) or 13.03 points (hip fracture patients that did not undergo inpatient rehabilitation after acute treatment), further underlining the importance of inpatient rehabilitation to ameliorate ADL in the long term [19].

Mobility scores at follow-up were compared to the pre-hospital scores; we observed a decent but not dramatic decline for both fracture groups.

There were neither significant nor tendentious differences between the major and minor groups concerning functional outcomes. Although minor non-immobilizing fractures were associated with a higher total BI at any time, no significantly different changes in BI or PMS were recorded from admission to follow-up. Immobilizing injuries were thus not associated with poorer functional outcomes. Gosch et al. showed comparable results, having observed a slight decline of PMS and a regain of BI without significant differences between hip-fracture and non-hip-fracture patients [7].

The length of stay was, on average 3.3 days longer after major than minor fractures, which is partially explained by the higher proportion of patients in this group receiving the total length of early inpatient rehabilitation (CEGH) of at least 16 days. The difference remains significant after correction for this confounding variable. This aligns with the clinical experience that lower extremity fractures require a more prolonged acute hospital stay consisting of rehabilitation and the organization of further social provisions than upper extremity fractures and thus supposedly were more often recommended for CEGH. As previously mentioned, the rehabilitative and therapeutic strategies were not different for non-CEGH patients. That the change in functional outcome parameters was not different between major and minor fractures confirms the value of a longer LOS after major fractures. Likewise, the literature reports a lower LOS to treat humeral fractures in geriatric patients [20, 21] and a higher LOS to treat hip fractures [9, 22].

The readmission rates were also comparable to reports in the literature [9]. Again, no difference was found between major or minor fractures, including for readmissions due to falls, which is interesting as one would suppose a lower extremity fracture would more strongly predispose for frequent falling than an upper extremity fracture would. This suggests that a fracture event, independent of the fracture site, is more of an indicator of general fall susceptibility. Furthermore, minor fractures can also cause immobility. Upper extremity fractures could result in unsteadiness in walking as the majority of geriatric patients cannot walk sufficiently and safely without aids like wheeled walkers. Additionally, even after rib fractures, a significant loss of self-care capacity and consequences like pneumonia or a predisposition for further falls or readmissions are conceivable [23, 24].

These observations assume that the primary goal for any injured patient (major or minor) should be the preservation of their daily living activities and mobility, which can more reliably be achieved with a multidisciplinary approach. This affirms that patients suffering from minor fractures should receive orthogeriatric co-management no less than those suffering from major fractures.

Care level and place of residence can be seen as surrogate parameters for, on one hand, the patient’s need for care and, on the other hand, socioeconomic elements. More than one-third of all patients were assessed at a higher care level at follow-up than before, with the minor group experiencing this to a significantly lower extent than the major group. Nevertheless, almost 30% of patients suffering from minor fractures experienced an upgrade in care levels, which is not a negligible proportion. This once again emphasizes the relevance of any fracture event to a patient’s self-support capability and its impact on health care systems.

This is also supported by the POR data, as almost one-fifth of all patients had to move (or leave their homes) permanently by the follow-up, with a tendentious, insignificantly lower rate in the minor fracture group. Additionally, about half of all patients that still lived at home needed more assistance from either family or care providers.

The personal evaluation of the situation at follow-up displays almost a normal distribution for all patients. Our results suggest better perceived conditions after minor fractures than after major ones.

Compared with our major group, Neuerburg et al. included only hip fractures and observed a similar distribution in their analogous assessment of the patient’s perceived “status of health” after orthogeriatric treatment. The results of a control group that was conventionally treated were significantly worse [9]. Shyu et al. also showed a significantly better “health-related quality of life” after interdisciplinary treatment of patients with fractured hips [25]. The literature provides some evidence in the field of hip fractures; a comparison or examination of other fragility fractures has not yet occurred. In this study, the major group included significantly more multimorbid individuals than the minor group, which could have exerted a nonnegligible confounding effect on the perceived evaluation. Consequently, we emphasize again the importance of understanding that minor fractures are not trivial events. Furthermore, by including the results from the literature, the relevance of orthogeriatric treatment for improvements in self-perceived health status is supported.

Concerning mortality, in-house and 1-year-mortality were significantly higher after major injuries than after minor; we observed no deaths in the minor group during the in-hospital course. Nevertheless, after 2 years, the significant difference in mortality had vanished. The persisting group-dependent difference might be further mitigated as the major group has a higher proportion of multimorbid patients. As previously mentioned in discussing the functionality data, this contradicts the presumption of overall higher death rates after major injuries (hip, thoracolumbar vertebral, pelvic, and other lower extremity fractures) than after minor injuries (upper extremity, cervical spine, and rib fractures). This is relevant to the comparison of any other outcome parameter assessed at follow-up, because higher mortality in one of the groups after 2 years could create a mortality bias, which our analysis did not discover. In summary, even mortality underlines a certain equality of outcomes with increasing observation time. Accordingly, Gosch et al. found no significant differences in mortality after hip and non-hip fractures for long-term care residents [7]. Contrarily, Center et al. describe remarkably higher survival rates after minor injuries. We must consider that patients in the mentioned study were included at the age of 60 and minor and major fractures were classified differently than in our study [26]. Bliuc et al., on the other hand, describe a relevant association of “nonhip nonvertebral fractures” and increased mortality [27]. As already mentioned, pain and immobility due to the inability to use aids might play a role in these results. Also, a general fracture event, whether major or minor, may be considered more of an overall indicator of higher morbidity and a higher probability for death, as a previous publication posited.

In general, the literature confirms that orthogeriatric treatment leads to improved survival after hip fractures compared with conventional treatment [6, 8, 9], and randomized, controlled trials for minor fractures could reveal similar results, as our observations suggest.

### Limitations and strengths

The overall cohort is comparable to existing studies [7, 9, 28]; nevertheless, our loss to follow-up was, unfortunately, not negligible, especially considering further losses in each parameter that made respective comparisons more difficult. We have reported many important outcome parameters of orthogeriatric treatment, although we have omitted some issues as stated in the “standard set of outcome parameters for the evaluation of orthogeriatric co-management” by Liem et al. [16]. This particularly concerns complications, which have been detailed in a previous publication. We did not examine a control group of non-orthogeriatric co-managed patients, nor did we examine any specific fracture for respective results due to insufficient remaining case numbers, which would have impaired statistical evaluation. Our approach, nevertheless, offers good opportunities for the comparison of major immobilizing injuries and minor non-immobilizing injuries, which is unique in itself. As previously explained, we have not estimated a relevant mortality bias for comparing the major and minor groups but a general mortality bias can be supposed for the overall outcome measurements and must be considered for comparisons to the literature.

As previously mentioned, the significantly higher number of multimorbid patients in the major group must also be considered as a biasing factor. We must, therefore, suppose a more equivalent outcome as more multimorbid patients are more likely associated with worse outcomes [28, 29]. In addition, treatment bias could have played a role in the actual results. Only patients who needed admission and met the admission criteria were included. As geriatric patients with major fractures are likely to be hospitalized, those suffering from minor fractures could have also been treated as outpatients and, consequently, have been omitted from the analysis, which could have had exacerbated our results regarding the minor group’s outcomes. Nevertheless, the admission score allowed some matching of both fracture groups and could have reduced the effects of other confounding factors. Lastly, the different treatments (different surgical treatment strategies and conservative vs. operative treatment) must also be considered as possible confounders of the outcomes.

## Conclusion

In this prospective 2 year follow-up on orthogeriatric co-management, we observed comparable outcomes for the functional and socioeconomic aspects of major and minor injuries.

These results underline the importance of orthogeriatric care in the treatment of fragility fractures and highlight the equal severity of any injury. Minor fractures should receive multidisciplinary care to the same extent as major fractures and their severity should not be underestimated. Upcoming investigations in the orthogeriatric field should assess more than just hip fractures so the most common and more rare fragility fractures can be analyzed analogously to either support or relativize our findings.

## Supplementary Information

Below is the link to the electronic supplementary material.

Supplementary file1 (DOCX 16 KB)

## Data Availability

The datasets used and analyzed in this study are available from the corresponding author on reasonable request.

## Abbreviations

ADL: Activities of daily living
BI: Barthel index
CEGH: Complex early geriatric rehabilitation
CL: Care level
HR: Hazard ratio
LOS: Length of stay
MaF: Major fractures
MiF: Minor fractures
PMS: Parker mobility score
POR: Place of residence

## References

[1] Borgström F, Karlsson L, Ortsäter G, Norton N, Halbout P, Cooper C, Lorentzon M, McCloskey EV, Harvey NC, Javaid MK, Kanis JA, Reginster JY, Ferrari S (2020). Fragility fractures in Europe: burden, management and opportunities. Arch Osteoporos..

[2] Pioli G, Bendini C, Pignedoli P, Giusti A, Marsh D (2018). Orthogeriatric co-management – managing frailty as well as fragility. Injury..

[3] Kammerlander C, Roth T, Friedman SM, Suhm N, Luger TJ, Kammerlander-Knauer U, Krappinger D, Blauth M (2010). Ortho-geriatric service-a literature review comparing different models. Osteoporos Int.

[4] Moyet J, Deschasse G, Marquant B, Mertl P, Bloch F (2019). Which is the optimal orthogeriatric care model to prevent mortality of elderly subjects post hip fractures? A systematic review and meta-analysis based on current clinical practice. Int Orthop.

[5] Grigoryan KV, Javedan H, Rudolph JL (2014). Orthogeriatric care models and outcomes in hip fracture patients: a systematic review and meta-analysis. J Orthop Trauma..

[6] Baroni M, Serra R, Boccardi V, Ercolani S, Zengarini E, Casucci P, Valecchi R, Rinonapoli G, Caraffa A, Mecocci P, Ruggiero C (2019). The orthogeriatric comanagement improves clinical outcomes of hip fracture in older adults. Osteoporos Int.

[7] Gosch M, Hoffmann-Weltin Y, Roth T, Blauth M, Nicholas JA, Kammerlander C (2016). Orthogeriatric co-management improves the outcome of long-term care residents with fragility fractures. Arch Orthop Trauma Surg.

[8] Rapp K, Becker C, Todd C, Rothenbacher D, Schulz C, König H-H, Liener U, Hartwig E, Büchele G (2020). The association between orthogeriatric co-management and mortality following hip fracture. Dtsch Aerzteblatt Online.

[9] Neuerburg C, Förch S, Gleich J, Böcker W, Gosch M, Kammerlander C, Mayr E (2019). Improved outcome in hip fracture patients in the aging population following co-managed care compared to conventional surgical treatment: a retrospective, dual-center cohort study. BMC Geriatr.

[10] Knobe M, Böttcher B, Coburn M, Friess T, Bollheimer LC, Heppner HJ, Werner CJ, Bach J-P, Wollgarten M, Poßelt S, Bliemel C, Bücking B (2019). AltersTraumaZentrum DGU®: evaluation klinischer und ökonomischer Parameter. Unfallchirurg.

[11] Schray D, Ehrnthaller C, Pfeufer D, Mehaffey S, Böcker W, Neuerburg C, Kammerlander C, Zeckey C (2018). Outcome after surgical treatment of fragility ankle fractures in a certified orthogeriatric trauma center. Injury.

[12] Adam J, Basil Ammori M, Isah I, Jeyam M, Butt U (2020). Mortality after inpatient stay for proximal humeral fractures. J Shoulder Elb Surg.

[13] Mahoney FI, Barthel DW (1965). Functional evaluation: the barthel index. Md State Med J.

[14] Parker MJ, Palmer CR (1993). A new mobility score for predicting mortality after hip fracture. J Bone Joint Surg Br.

[15] Charlson ME, Pompei P, Ales KL, MacKenzie R (1987). A new method of classifying prognostic in longitudinal studies: development and validation. J Chronic Dis.

[16] Liem IS, Kammerlander C, Suhm N, Blauth M, Roth T, Gosch M, Hoang-Kim A, Mendelson D, Zuckerman J, Leung F, Burton J, Moran C, Parker M, Giusti A, Pioli G, Goldhahn J, Kates SL (2013). Identifying a standard set of outcome parameters for the evaluation of orthogeriatric co-management for hip fractures. Injury.

[17] Prestmo A, Hagen G, Sletvold O, Helbostad JL, Thingstad P, Taraldsen K, Lydersen S, Halsteinli V, Saltnes T, Lamb SE, Johnsen LG, Saltvedt I (2015). Comprehensive geriatric care for patients with hip fractures: a prospective, randomised, controlled trial. Lancet..

[18] Stenvall M, Olofsson B, Nyberg L, Lundström M, Gustafson Y (2007). Improved performance in activities of daily living and mobility after a multidisciplinary postoperative rehabilitation in older people with femoral neck fracture: a randomized controlled trial with 1-year follow-up. J Rehabil Med..

[19] Pfeufer D, Kammerlander C, Stadler C, Roth T, Blauth M, Neuerburg C, Böcker W, Zeckey C, Lechleitner M, Gosch M (2020). Multidisciplinary inpatient rehabilitation improves the long-term functional status of geriatric hip-fracture patients. Eur J Med Res.

[20] Wang MQ, Youssef T, Smerdely P (2018). Incidence and outcomes of humeral fractures in the older person. Osteoporos Int..

[21] Lander ST, Mahmood B, Maceroli MA, Byrd J, Elfar JC, Ketz JP, Nikkel LE (2019). Mortality rates of humerus fractures in the elderly: does surgical treatment matter?. In: J Orthopaedic Trauma.

[22] Aletto C, Aicale R, Pezzuti G, Bruno F, Maffulli N (2020). Impact of an orthogeriatrician on length of stay of elderly patient with hip fracture. Osteoporos Int..

[23] Halevi AE, Mauer E, Saldinger P, Hagler DJ (2018). Predictors of dependency in geriatric trauma patients with rib fractures: a population study. Am Surg.

[24] Mai HT, Tran TS, Ho-Le TP, Pham TT, Center JR, Eisman JA, Nguyen TV (2018). Low-trauma rib fracture in the elderly: risk factors and mortality consequence. Bone..

[25] Shyu YIL, Liang J, Wu CC, Cheng HS, Chen MC (2010). An interdisciplinary intervention for older Taiwanese patients after surgery for hip fracture improves health-related quality of life. BMC Musculoskelet Disord.

[26] Center JR, Nguyen TV, Schneider D, Sambrook PN, Eisman JA (1999). Mortality after all major types of osteoporotic fracture in men and women: an observational study. Lancet..

[27] Bliuc D, Nguyen ND, Milch VE (2010). Mortality risk associated with low-trauma fracture in men and women. Jama.

[28] Folbert EC, Hegeman JH, Vermeer M, Regtuijt EM, van der Velde D, ten Duis HJ, Slaets JP (2017). Improved 1-year mortality in elderly patients with a hip fracture following integrated orthogeriatric treatment. Osteoporos Int..

[29] Wiedl A, Förch S, Fenwick A, Mayr E (2021). Prognostic value of orthogeriatric assessment parameters on mortality: a 2-year follow-up. Eur J Trauma Emerg Surg..

