# Supplementary material for: Orthogeriatric co-management: differences in outcome between major and minor fractures
Source: Eur J Trauma Emerg Surg. 2022 Apr 28;48(4):2953–66. doi: 10.1007/s00068-022-01974-3 (PMC9360167; doi:10.1007/s00068-022-01974-3)
Supplement: Supplementary file 1 — Supplementary file1 (DOCX 16 KB) [file 68_2022_1974_MOESM1_ESM.docx]

| Supplemental Table 1 Assessment rates of each assessment variate in percentages, total numbers in brackets. References for follow-up parameters after correction for loss to follow-up and deceased patients | | | |
| --- | --- | --- | --- |
|  | All patients | Major fractures | Minor fractures |
| CCI | 98.4%(728) | 98.6%(566) | 97.6%(162) |
| BI on admission | 95.1% (704) | 94.9% (545) | 95.8% (159) |
| BI on discharge | 88.1% (652) | 89.2% (512) | 84.3% (140) |
| BI in Follow-up Group | 98.3% (349) | 98.1% (259) | 89.9% (90) |
| PMS before admission | 86.5% (640) | 86.4% (496) | 86.7% (144) |
| PMS in Follow-up Group | 98.6% (350) | 98.5% (260) | 98.9% (90) |
| Admission from | 97.2% (719) | 97.2%(558) | 97.0% (161) |
| POR at Follow-up | 99.4% (353) | 99.2% (262) | 100%(91) |
| Care level before admission | 46.1% (341) | 44.1% (253) | 53.0% (88) |
| Care level at Follow-up | 96.1% (341) | 95.8% (253) | 96.7% (88) |
| Subjective impression of situation | 90.4% (321) | 89.4% (236) | 93.4% (85) |
| Rehospitalization | 81.8% (323) | 90.9% (240) | 91.2% (83) |
